# Changes in Health Indicators of Welfare in Group-Housed Shelter Cats

**DOI:** 10.3389/fvets.2021.701346

**Published:** 2021-09-24

**Authors:** Veronika Vojtkovská, Eva Voslářová, Vladimír Večerek

**Affiliations:** Department of Animal Protection and Welfare and Veterinary Public Health, University of Veterinary Sciences Brno, Brno, Czechia

**Keywords:** stray animal, shelter, welfare, animal-based indicators, group housing

## Abstract

The aim of this study was to detect changes in health-oriented welfare indicators of shelter cats housed in a shelter by means of long-term monitoring of health indicators of welfare in a population of group-housed cats in a private, no-kill shelter in the Czech Republic. The cat population housed in a large group was monitored for 1 year. The data recording took place at 2-week intervals. A total of 220 cats were evaluated using a protocol containing eight health-oriented welfare indicators: body condition, third eye visibility, eye discharge, eye irritation, nose discharge, the presence of pathologically induced respiratory sounds, coat condition, and lameness and abnormal posture. The assessment was performed based on the observation by two trained evaluators. The cats' condition was rated on a 5-point scale for each indicator, where the optimal condition was represented by the score of 1 and a severe deterioration by the score of 5 for the given indicator, except for the body condition indicator, where the optimal condition was represented by the score of 3. A deterioration in the score in at least one of the indicators during the stay in the shelter was recorded in 52 (41.6%) of 125 cats that were assessed at least twice. The effect of the LOS, sex and age on the scores for each health indicator was examined *via* a linear mixed model analysis, as this method allows for handling of dependencies in the data of repeated measurements. The effect of predictors on the third eye visibility scores was not found. The age of cats predicted the coat condition and body condition scores. The LOS predicted scores of the abnormal posture and lameness indicator and scores of a composite index composed of indicators related to upper respiratory tract disease. The results suggest that despite the fact that some improvement of health was documented during the cats' stay in the shelter, there were a non-negligible number of animals experiencing a permanent or long-term deterioration in health. Efforts to minimize the undesirable factors contributing to the deterioration of well-being of cats during their stay in a shelter should be made.

## Introduction

In general, the goal of the facilities providing care for abandoned and stray animals is not only to ensure as many adoptions as possible and to return the animals to their original owners but also to provide a temporary accommodation that meets the animals' requirements in terms of suitable nutrition, housing, healthcare, and human contact. Shelters are not facilities capable of providing an ideal long-term environment for animals and they do not fully substitute a new home (1); the quality of care provided is a critical aspect of welfare in many shelters (2). The chances of meeting the natural requirements of cats can vary considerably across different types of facilities (3). Some countries (e.g., the Czech Republic, Austria, Italy, Germany and Greece) (1, 4, 5) have implemented no-kill shelter policies in their national legislation. In these shelters, an animal cannot be euthanized due to shelter overpopulation or due to the animal's low adoption appeal to potential new owners, which is typically the case of older and handicapped cats (6). A long-term stay in a shelter may pose a risk to the animal's welfare reflected in the animal's behavior (animals may face long-term stress) and deterioration in health (1).

The environmental factors of the shelter (placement into an unfamiliar environment sometimes lacking stimuli, a lack of space, change of daily routine, veterinary treatment, presence of other animals and unfamiliar humans, and a general lack of control over the environment) can be perceived as stressful by a cat (7–9). Stress affects cats' well-being through inhibition of some behaviors such as food intake, elimination, grooming, exploration, and play. With increasing level of stress, cats increase the frequency and intensity with which they attempt to hide (2). Behavioral changes associated with chronic stress may increase the risk of euthanasia and reduce adoptability (10). Earlier studies documented the association between stress and impaired immunity resulting in the development or reoccurrence of diseases (11–13). Stress, for instance, play an important role in the reactivation of feline herpesvirus (14). Stress has been also associated with several gastrointestinal problems such as diarrhea or vomiting (15) and it can contribute to the development of feline interstitial cystitis, which is the most common diagnosis in cats with feline lower urinary tract disease (16). Furthermore, there is a clear connection between skin and nervous system (17), atopic diseases or acral lick dermatitis may be affected by stress. Stress is likely to trigger or perpetuate pruritus (18). In addition to long-term stress, however, the health of cats can be endangered by the presence of cats with an unknown medical history. A higher concentration of animals is associated with higher morbidity rates (19). From the viewpoint of disease control and prevention, forming large groups is not recommended due to a high turnover of animals (19); despite that, large groups are formed in many shelters, mainly due to a lack of space (6). Across countries, the preferred type of shelter cat housing is in groups. The advantages and disadvantages of group housing, as opposed to individual housing, are being studied by the scientific community (20–26). Considering that the cat's health may be impaired after the admission into a shelter, adequate health indicators should be included in the welfare assessment protocol of shelters where animals may be housed for extended periods due to the no-kill policy (27). The selected indicators should reflect the most commonly observed signs of impaired health in cats in shelters (1). Frequently occurring health issues include the upper respiratory tract disease (URTD) (13, 28), which is commonly caused by feline calicivirus (FCV) and feline herpesvirus (FHV-1) (29). Therefore, it is appropriate to assess the specific symptoms (e.g., discharge from the eyes and nose) (13). Examining the body condition can provide useful information, as changes in the body weight may indicate a severe medical condition or the presence of stress in general (13, 30). Inadequate grooming behavior, malnutrition, social conflicts in the group, and chronic diseases are reflected in the coat quality, so it should also be included among the welfare indicators (31), as well as the inability of the animal to move normally due to an injury or a disease (32, 33).

The primary aim of our study was long-term monitoring of changes in selected health-oriented welfare indicators of cats in a private no-kill shelter where animals are housed together in one large group. The secondary goal was to determine whether staying in a shelter causes an improvement or deterioration in the health condition of cats. We presumed that changes in the scores in health indicators of cats during their stay in the shelter would occur. Furthermore, since the length of stay of cats in the shelter vary and the cats staying shorter than 2 weeks could be assessed only once, we aimed to analyze also the results of the first assessment upon admission in order to include all cats admitted during the monitored period and to provide information on general health status of cats admitted to the shelter.

## Materials and Methods

### Study Site

The data collection was carried out in a private shelter with group-housed cats located in the Czech Republic. The shelter adheres to the no-kill policy, as euthanizing an animal for reasons other than a health impairment is not permitted in the Czech Republic (Act No 246/1992 Coll., on the protection of animals against cruelty, as amended). The capacity of the monitored shelter is 25 cats, with an annual admission rate of ~200 cats. After admission to the shelter, all cats were placed in separate quarantine units (quarantine boxes with the dimensions of 80 × 40 × 60 cm furnished with a cat toilet, bowls with food and water, a toy, and a cat bed) individually or in a group (in case of the cats admitted from the same source). The shelter has a total of 5 quarantine boxes available. While in quarantine, all cats were inspected by a contracted veterinarian and the basic veterinary procedures were performed (vaccination, internal and external parasite treatment, and neutering) with respect to the animal's age and health. The length of the quarantine period depended on the health status of each cat, however, it did not last <5 days in any case. After the quarantine period, the cats were released into a group of other cats. A total area of 53 m^2^ is used for housing cats. The shelter consists of two rooms (38 m^2^) and an outdoor pen (15 m^2^) and these areas are interconnected. All cats housed in the shelter have a year-round access to the outdoor pen. The quarantine boxes, the examination table, the equipment for food preparation and medical treatment of the animals are placed in the first room. Within the interior of the shelter, there are a total of eight cat toilets, five bowls of dry food, seven bowls of water, toys, and other objects in which the cats can hide or rest (beds and cat trees). This area is connected with the outdoor pen *via* a cat door. The described spaces accessible to cats cannot be subdivided, which prevents the shelter from forming homogeneous groups (housing cats of a similar age or cats admitted from one household). The exceptions are cats with offspring and cats in advanced stages of gestation, for which the shelter has separate premises. Only one mother with offspring may be placed in this area. In the case of admitting another litter of offspring, the animals are relocated to another shelter due to capacity constraints. The animals are attended by one caregiver; in addition to the dry food that is still available, the cats are fed wet food twice a day at five sites. Wet food was provided to the cats under supervision to ensure all cats received a sufficient amount of food and had access to it. Only complete super premium cat food of various brands was used for feeding. The cat toilets are cleaned daily or as needed; the flooring of the shelter is disinfected routinely every 2 days.

### Assessment of Cats' Health

**Figure 1:**
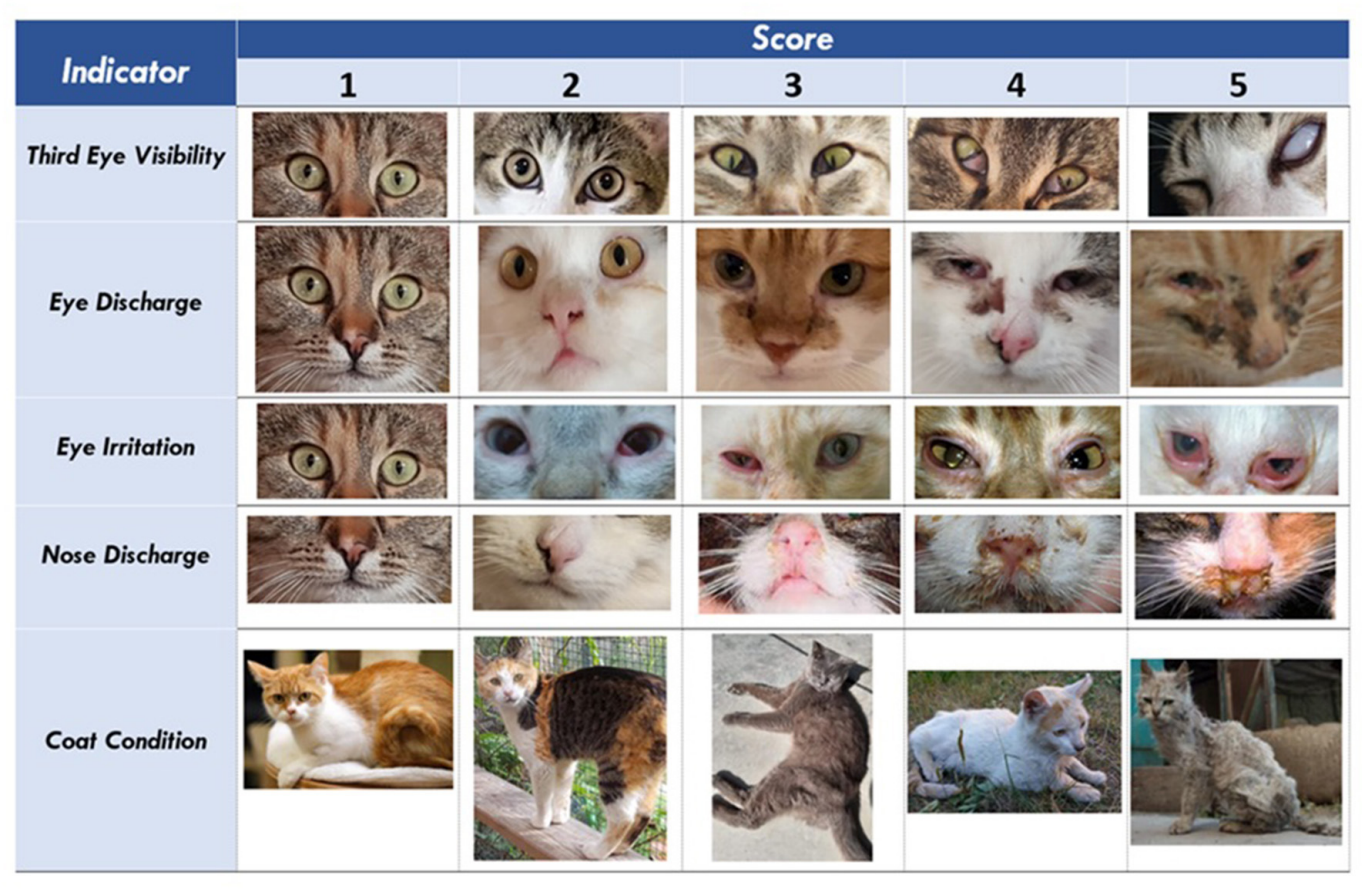
Calibration photo sheet—assessment of cats.

Based on available scientific knowledge (1, 7, 34, 35), consultations and preliminary screening for signs of deterioration in cats in the selected shelter, a protocol consisting of eight visually assessable, health-oriented welfare indicators was designed in cooperation with a member of the shelter staff and a veterinarian specializing in feline medicine. The indicators included in the protocol were selected to reflect the most frequently observed signs of deteriorating health of the cats in the shelter. To record even smaller deviations in the condition of cats in the individual indicators, a 5-point scale was designed. A score of 1 represented the best possible condition, while the score of 5 a serious deterioration. A 5-point body condition score (BCS) scale by Shoveller et al. (36) with the optimum score of 3 was used to assess the body condition of cats. A more detailed specification of the included indicators and a description of the scores are summarized in Table 1. To ensure the consistency of the assessment and to facilitate the assessment procedure, the calibration sheet consisting of images with the individual scores of the indicators was used (Figure 1).

**Table 1:** Assessment protocol—description of scores in individual indicators.

**Indicator**	**Assessment specification**	**Score 1**	**Score 2**	**Score 3**	**Score 4**	**Score 5**
Body condition	Amount of body fat	Very low (thin)	Low (underweight)	Moderate (ideal)	High (overweight)	Very high (obese)
Third eye visibility	Intensity of visibility of the third eyelid	Absent	Weak	Moderate	Strong	Complete
Eye discharge	Discharge intensity	None discharge	Weak discharge	Moderate discharge	Strong discharge	Very strong discharge
Eye irritation	Redness, swelling	None eye irritation	Weak irritation	Moderate irritation	Strong irritation	Very strong irritation
Nose discharge	Discharge intensity	None discharge	Weak discharge	Moderate discharge	Strong discharge	Very strong discharge
Presence of pathologically induced respiratory sounds	Frequency of sounds—coughing, sneezing, and coarse crackles	No pathological sounds	Pathological sounds occur rarely	Pathological sounds occur occasionally	Pathological sounds occur often	Pathological sounds occur very often
Coat condition	Coat quality and skin condition	Excellent condition	Good condition	Fair condition	Poor condition	Very poor condition
Abnormal posture and lameness	Ease of movement and walking	No abnormal posture or lameness; cat moves easily	Cat is lightly lame or shows lightly abnormal posture	Cat is moderately lame or shows moderately abnormal posture	Cat is seriously lame or shows serious abnormal posture	Cat is unable to walk or move due to abnormal posture or lameness

Some health indicators could not be included in the assessment protocol due to the group housing of cats. Elimination-related indicators such as diarrhea and symptoms of a lower urinary tract disease (37, 38) were not assessed as the animals shared toilets, which made it impossible to identify the specific individuals with a problem without constant animal supervision. In addition, the control of a selected animal suspected of having a problem can be quite difficult, as cats often refuse to excrete when disturbed by the presence of people (39).

During the assessment, the cats were not handled. All animals were evaluated visually. The cats that rested or played together were assessed at the same time. In case of the evaluation of the presence of pathological sounds, each animal was observed for a half an hour by an observer standing in the proximity of the cat assessed. Fearful animals that did not allow to be assessed from close proximity were evaluated from the distance they found comfortable. The distance between a fearful cat and the evaluator was different for each animal. However, in no case was the distance too far to prevent the animal from being properly evaluated.

To detect changes in the selected indicators even in animals staying in the shelter for an extended period, the population was monitored for 12 months (beginning in March 2019, ending in March 2020). The data recording (assessing the cats in each indicator) took place at two-week intervals at the same time (the evaluation always started at 6 p.m.). The score rated in each of the indicators represented the condition of the cat for the given indicator at the time of monitoring. The score always ranged from 1 to 5. The monitoring was conducted on all individuals staying in the shelter at the time. The data collection was carried out only in newly admitted animals (cats staying in the shelter prior to the start of the data collection were not included in the study). Animals housed in the shelter before the start of data collection were excluded as their health status and any changes to their health occurring during their stay in the shelter before the monitored period could not be evaluated. For the same reason, also the cats that remained in the shelter after the end of the monitored period were excluded; subsequent changes in their health potentially occurring during their stay in the shelter after the monitoring not evaluated. During the monitored period, a total of 220 cats were assessed (all cats were assessed at least once after the admission to the shelter). The maximum number of assessments was 14, thus no cat stayed in the shelter for more than 26 weeks. Additional information for each assessed cat (sex, age, breed, date of admission, date of the termination of the stay in the shelter and outcome) were obtained from the shelter records. The assessment of cats was carried out by a researcher specialized in cat welfare assessment (the author of this study) in cooperation with a trained caretaker of shelter cats. No formal statistical testing of inter-and intra-rater reliability was conducted. However, an informal intra-observer reliability calibration was performed during the assessment. This was done *via* discussion between both evaluators about each cat until an agreement reached.

### Statistical Analysis

#### χ^2^ Test, Correlation Analysis

The χ^2^ test and the correlation analysis were performed by statistical software Unistat 6.5 for Excel (Unistat Ltd., UK). A value of *P* ≤ 0.05 was considered statistically significant. The Shapiro–Wilk test was used to verify the normal distribution of the data (an irregular distribution was detected). The differences in the number of animals in optimal and suboptimal conditions, the number of cats with changed and unchanged scores and improved and worsened scores were analyzed by the χ^2^ test (2 × 2 contingency tables). A Yates correction was used on frequencies exceeding 5. The Fisher's exact test was used at frequencies lower than 5. The Spearman's rank correlation coefficient was used to verify the relation between the mean scores of cats among the individual indicators. For the purposes of establishing the relationship between the mean scores of the body condition indicator and other indicators, the 5-point body condition scale was transformed into a 3-point system, where the score of 3 represented the optimal condition (ideal body condition), the score 2 a slight deterioration (underweight and overweight) and the score 1 a significant deterioration (thin or obese). This procedure was followed as the body condition scale is not normally rated in the form of a continuum where the highest score represents the best or worst possible condition.

#### Linear Mixed Model

The linear mixed models were performed in the statistical program JASP 0.14.1. A value of *P* ≤ 0.05 was considered statistically significant. The Satterthwaite's method was used to estimate the degrees of freedom and generate *P*-values for mixed models. The effect of the length of stay (LOS, calculated in days as the difference between the cat's intake date and the date when its stay in the shelter was terminated), sex (male vs. female), and age (≤6 months; 6 < × ≤ 12 months; 1 < × ≤ 8 years; >8 years—sum contrast coded) on scores for each health-related indicator was examined *via* the linear mixed model analysis as this method allows for handling of dependencies in the data of repeated measurements. Our model consisted of both, fixed and mixed effects. The cats were treated as nested random factors. The predictors of interest were treated as fixed factors. For this analysis, we used four more specific outcome variables—the evaluation of third eye visibility, lameness and abnormal posture, coat condition, and body condition as well as one more complex composite indicator (CCI) composed of eye discharge, eye irritation, nose discharge, and the presence of pathological sounds. These indicators were combined into one composite index as they covered conceptually similar symptoms and established high inter-correlations. The internal consistency for the scale was good (Cronbach alpha = 0.72).

## Results

### Characteristics of the Monitored Cat Population

A total of 220 cats were assessed during the monitored period; the median LOS of cats in the shelter was 40 days (range = 1–186 days). Table 2 shows the number of cats assessed during each week. The characteristics of the monitored population of shelter cats are presented in Table 3.

**Table 2:** Number of cats assessed in the shelter during each week of assessment.

**Number of ratings and a corresponding number of weeks the cat has stayed in the shelter**	**Number of cats that spent the given number of weeks in the shelter**	**Total number of cats that were assessed at least the given number of times**
1 assessment = 0 weeks in shelter	82	220
2 assessments = 2 weeks in shelter	43	138
3 assessments = 4 weeks in shelter	39	95
4 assessments = 6 weeks in shelter	23	56
5 assessments = 8 weeks in shelter	8	33
6 assessments = 10 weeks in shelter	7	25
7 assessments = 12 weeks in shelter	5	18
8 assessments = 14 weeks in shelter	1	13
9 assessments = 16 weeks in shelter	4	12
10 assessments = 18 weeks in shelter	2	8
11 assessments = 20 weeks in shelter	1	6
12 assessments = 22 weeks in shelter	2	5
13 assessments = 24 weeks in shelter	0	3
14 assessments = 26 weeks in shelter	3	3

**Table 3:** Characteristics of cats assessed in the shelter (sex, age, and breed).

	***N***	**%**
**Sex**		
Males	87	39.5
Females	133	60.5
**Age**		
≤6 months	112	50.9
6 < × ≤ 12 months	41	18.6
1 < × ≤ 8 years	59	26.8
>8 years	8	3.6
**Breed**		
Domestic shorthair	188	85.5
Domestic longhair	8	3.6
Purebred	24	10.9
**Outcome**		
Adoption	154	70.0
Died/euthanized	49	22.3
Captured and released	2	0.9
Reclaimed	2	0.9
Stayed in the shelter	13	5.9

### Results of the Health Assessment of the Cats Upon Admission to the Shelter

Out of the total of 220 cats, upon admission, a worsened condition (a score of 2 or greater rated in at least one indicator, or a score of 1, 2, 4, and 5 in the body condition indicator) was found in 120 animals (54.5%). One hundred cats (45.5%) were found in an optimal condition (a score of 3 in the body condition indicator and a score of 1 in all other indicators).

The results of the assessment of the condition of the cats upon admission to the shelter for the individual health indicators are summarized in Table 4. In all indicators, the difference between the number of admitted cats with optimal scores (a score of 3 in the body condition indicator and a score of 1 in all other indicators) and the number of cats with impaired scores (a score of 2–5, or a score of 1, 2, 4, and 5 in the case of the body condition indicator) was found significant (*P* < 0.001).

**Table 4:** Number of cats that were rated specific scores in individual indicators upon admission to the shelter.

**Indicator**	**Score 1 (%)**	**Score 2 (%)**	**Score 3 (%)**	**Score 4 (%)**	**Score 5 (%)**
Body condition	2 (0.9)	60 (27.3)	137 (62.3)	20 (9.1)	1 (0.5)
Third eye visibility	209 (95.0)	9 (4.1)	1 (0.5)	0 (0.0)	1 (0.5)
Eye discharge	182 (82.7)	25 (11.4)	7 (3.2)	5 (2.3)	1 (0.5)
Eye irritation	195 (88.6)	15 (6.8)	6 (2.7)	2 (0.9)	2 (0.9)
Nose discharge	205 (93.2)	10 (4.5)	4 (1.8)	1 (0.5)	0 (0.0)
Presence of pathologically induced respiratory sounds	197 (89.5)	12 (5.5)	10 (4.5)	1 (0.5)	0 (0.0)
Coat condition	187 (85.0)	15 (6.8)	17 (7.7)	1 (0.5)	0 (0.0)
Abnormal posture and lameness	208 (94.5)	4 (1.8)	2 (0.9)	2 (0.9)	4 (1.8)

### Results of the Assessment of Changes in Health Indicators During the Cats' Stay in the Shelter

A deterioration in the score in at least one of the indicators during the stay in the shelter was recorded in 52 (41.6%) of the 125 cats that were assessed at least twice (and their stay in the shelter was terminated during the monitored period). On the other hand, in 37 cats (29.6%), there was no change in the score in any indicator during the stay compared to the score rated upon admission. Among them, 33 cats maintained the optimal score (a score of 3 in the body condition indicator and a score of 1 in all other indicators) in all indicators during the entire stay in the shelter.

Out of the 125 cats assessed at least twice and having left the shelter during the monitored period, 66 cats (52.8%) were in an optimal condition (a score of 3 in the body condition indicator and a score of 1 in all other indicators) in their last assessment, while 59 cats (47.2%) were rated with a score of 2 or worse in at least one of the indicators (or a score of 1, 2, 4, and 5 in the body condition indicator). No significant difference (*P* = 0.4479) was found between the number of cats in an optimal and suboptimal condition in their last assessment. Likewise, no significant difference (*P* = 0.1286) was found when comparing the number of cats (*n* = 53) with the optimal score in the first assessment and cats (*n* = 66) whose condition was optimal in their last assessment. No significant difference was found when comparing number of cats whose condition was deteriorated on admission (*n* = 72) and in their last assessment (*n* = 59).

Table 5 shows the number of cats whose score changed (improved/worsened) or did not change in the individual indicators during their stay in the shelter. Only cats that were assessed at least twice were included. Cats that did not leave the shelter by the end of the monitored period were excluded from this analysis.

**Table 5:** Number of cats whose score in the individual indicators changed (worsened or improved; first worsened and then improved or first improved and then worsened) or did not change at any time during their stay in the shelter.

**Indicator**	^*^Score unchanged (%)^**A**^	^**^Score changed (%)^**B**^	***P*-value^**AB**^**	**^&^Score improved (%)^**C**^**	**^#^Score worsened (%)^**D**^**	***P*-value^**CD**^**	**^a^Score improved then worsened (%)^**E**^**	**^b^Score worsened then improved (%)^**F**^**	***P*-value^**EF**^**
Body condition	73 (58.4)	52 (41.6)	0.0114	30 (24.0)	10 (8.0)	0.0010	7 (5.6)	5 (4.0)	>0.05
Third eye visibility	117 (93.6)	8 (6.4)	<0.001	3 (2.4)	2 (1.6)	>0.05	1 (0.8)	2 (1.6)	>0.05
Eye discharge	88 (70.4)	37 (29.6)	<0.001	14 (11.2)	6 (4.8)	>0.05	2 (1.6)	15 (12.0)	0.0026
Eye irritation	98 (78.4)	27 (21.6)	<0.001	13 (10.4)	4 (3.2)	0.0445	2 (1.6)	8 (6.4)	>0.05
Nose discharge	108 (86.4)	17 (13.6)	<0.001	6 (4.8)	3 (2.4)	>0.05	3 (2.4)	5 (4.0)	>0.05
Presence of pathologically induced respiratory sounds	102 (81.6)	23 (18.4)	<0.001	11 (8.8)	2 (1.6)	0.0227	3 (2.4)	7 (5.6)	>0.05
Coat condition	102 (81.6)	23 (18.4)	<0.001	18 (14.4)	4 (3.2)	0.0037	0 (0.0)	1 (0.8)	>0.05
Abnormal posture and lameness	119 (95.2)	6 (4.8)	<0.001	5 (4.0)	1 (0.8)	>0.05	0 (0.0)	0 (0.0)	>0.05

* *Number of cats whose score did not change throughout their entire stay in the shelter; the score remained the same throughout the entire stay*.

** *Number of cats whose score changed throughout their stay in the shelter compared to the score on admission*.

& *Number of cats whose score improved throughout their stay in the shelter*.

# *Number of cats whose score deteriorated throughout their stay in the shelter*.

a *Number of cats whose score first improved and then deteriorated throughout their stay in the shelter*.

b *number of cats whose score first deteriorated and then improved throughout their stay in the shelter. The letters A–F were used as symbols to indicate the data that were compared by statistical analysis within the rows of the two columns*.

Correlations of the mean scores among the indicators are shown in Table 6. Indicators related to URTD (eye discharge, eye irritation, nose discharge, and the presence of pathologically induced respiratory sounds) correlated. In addition, a correlation between the body condition and the coat condition, the coat condition and some of the URDT indicators (the presence of pathologically induced respiratory sounds, eye discharge, and third eye visibility) was found.

**Table 6:** Correlations between mean scores of the individual indicators (Spearman's rank correlation coefficient and *P*-values).

**Indicator**	**Third eye visibility**	**Eye discharge**	**Eye irritation**	**Nose discharge**	**Presence of pathologically induced respiratory sounds**	**Coat condition**	**Lameness and abnormal posture**
Body condition	*r_*s*_* = −0.506; *P* > 0.05	*r_*s*_* = −0.0094; *P* > 0.05	*r_*s*_* = 0.0128; *P* > 0.05	*r_*s*_* = −0.0261; *P* > 0.05	*r_*s*_* = 0.0078; *P* > 0.05	*r_*s*_* = −0.2137; *P* = 0.0020	*r_*s*_* = 0.0670; *P* > 0.05
Third eye visibility	–	*r_*s*_* = 0.3609; *P* < 0.001	*r_*s*_* = 0.3233; *P* < 0.001	*r_*s*_* = 0.3359; *P* < 0.001	*r_*s*_* = 0.1018; *P* > 0.05	*r_*s*_* = 0.1712; *P* = 0.0137	*r_*s*_* = 0.0520; *P* > 0.05
Eye discharge	–	–	*r_*s*_* = 0.6614; *P* < 0.001	*r_*s*_* = 0.4702; *P* < 0.001	*r_*s*_* = 0.3733; *P* < 0.001	*r_*s*_* = 0.2082; *P* = 0.0026	*r_*s*_* = 0.0955; *P* > 0.05
Eye irritation	–	–	–	*r_*s*_* = 0.3359; *P* < 0.001	*r_*s*_* = 0.3743; *P* < 0.001	*r_*s*_* = 0.1344; *P* > 0.05	*r_*s*_* = 0.0402; *P* > 0.05
Nose discharge	–	–	–	–	*r_*s*_* = 0.5326; *P* < 0.001	*r_*s*_* = 0.0449; *P* > 0.05	*r_*s*_* = 0.0817; *P* > 0.05
Presence of pathologically induced respiratory sounds	–	–	–	–	–	*r_*s*_* = 0.1551; *P* = 0.0257	*r_*s*_* = 0.0579; *P* > 0.05
Coat condition	–	–	–	–	–	–	*r_*s*_* = 0.0743; *P* > 0.05

### Role of the Length of Stay, Sex, and Age of the Cats on the Health Indicators

We ran the Linear Mixed Model (LMM) to assess the role of the LOS (in days) in a shelter, sex (male vs. female) and age (≤6 months; 6 < × ≤ 12 months; 1 < × ≤ 8 years; >8 years—sum contrast coded) in the evaluation of the third eye visibility, lameness and abnormal posture, coat condition, and body condition as well as a CCI.

When the LMM analysis was conducted for the third eye visibility as a dependent variable, none of the predictors was significant. In particular, the LOS, *F*_(1,97.37)_ = 0.04, *P* = 0.84, sex *F*_(1,145.09)_ = 0.12, *P* = 0.73, and age *F*_(3,132.54)_ = 1.35, *P* = 0.26, did not predict the third eye visibility. However, when the CCI was analyzed, there was a significant effect of the LOS *F*_(1,129.28)_ = 9.46, *P* = 0.003. The impact of sex *F*_(1,203.70)_ = 3.783, *P* = 0.05, and age *F*_(1,183.76)_ = 0.329, *P* = 0.80, was not significant. This means that the LOS in the shelter positively predicted a higher score of CCI (a higher score means a worse condition; *b* = 0.003, *SE* = 0.001, and *P* = 0.003), but not age and sex. Similarly, when the lameness and abnormal posture indicator was analyzed, a significant effect of the LOS was found, *F*_(1,201.54)_ = 37.291, *P* < 0.001. The effect of sex, *F*_(1,255.61)_ = 0.156, *P* = 0.693, and age, *F*_(3,242.83)_ = 0.644, *P* = 0.58 was not significant. As in the previous case, the LOS in the shelter positively predicted the score of lameness and abnormal posture (*b* = 0.003, *SE* = 4.444e−4, and *P* < 0.001), but not sex and age. The situation was different when the coat condition and body condition were analyzed. There was a significant impact of age *F*_(3,171.60)_ = 2.767, *P* = 0.043, meaning that in comparison to younger cats, old cats had higher scores in the coat condition indicator. The effect of the LOS *F*_(1,186.60)_ = 1.239, *P* = 0.267 or sex *F*_(1,186.60)_ = 1.239, *P* = 0.267 were not significant. Similarly, when the body condition was analyzed as a dependent variable, a significant effect of the age was found, *F*_(3,169.587)_ = 7.26, *P* < 0.001. In comparison to young animals, older cats had a higher score in this indicator. The impact of the LOS, *F*_(1,183.65)_ = 0.48, *P* = 0.49, or sex *F*_(1,128.57)_ = 2.64, *P* = 0.11, was not significant. The fixed effect estimates^1^ across all examined models are shown in Table 7.

**Table 7:** Fixed effect estimates for each of the analyzed indicators.

**Dependent variable**	**Predictor**	**Estimate**	**SE**	**df**	***t*-value**	***p*-value**
Body condition	Intercept	2.88	0.06	175.80	46.66	<0.001
	Days	7.79e−4	4.79e−4	128.57	1.63	0.11
	Sex	0.02	0.03	183.65	0.69	0.49
	Age (1)	−0.19	0.06	164.22	−3.16	0.001
	Age (2)	0.05	0.07	166.29	0.65	0.51
	Age (3)	0.18	0.07	174.91	2.55	0.01
Third eye visibility	Intercept	1.05	0.04	138.08	29.08	<0.001
	Days	5.52e−5	2.78e−4	97.37	0.20	0.84
	Sex	7.05e−3	0.02	145.09	0.35	0.73
	Age (1)	−8.89e−3	0.03	127.88	−0.26	0.80
	Age (2)	0.08	0.04	129.65	1.93	0.06
	Age (3)	−0.03	0.04	137.33	−0.62	0.54
CCI (composed of eye discharge, eye	Intercept	4.44	0.15	192.93	29.50	<0.001
irritation, nose discharge, and presence of	Days	3.49e−3	1.14e−3	129.28	3.08	0.002
pathologically induced respiratory sounds)	Sex	0.17	0.09	203.70	1.94	0.05
	Age (1)	−0.05	0.14	177.30	−0.35	0.73
	Age (2)	0.10	0.18	179.21	0.53	0.60
	Age (3)	−0.14	0.17	192.23	−0.85	0.40
Coat condition	Intercept	1.29	0.05	178.27	24.03	<0.001
	Days	−5.55e−4	4.15e−4	128.39	−1.34	0.18
	Sex	0.03	0.03	186.60	1.11	0.27
	Age (1)	−0.14	0.05	166.03	−2.68	0.008
	Age (2)	−0.08	0.06	168.11	−1.22	0.22
	Age (3)	−0.04	0.06	177.40	−0.61	0.55
Lameness and abnormal posture	Intercept	0.87	0.06	243.40	13.53	<0.001
	Days	2.81e−3	5.12e−4	203.68	5.50	<0.001
	Sex	−3.25e−3	0.04	249.27	−0.09	0.93
	Age (1)	0.05	0.06	233.73	0.77	0.44
	Age (2)	0.03	0.08	236.12	0.34	0.73
	Age (3)	0.07	0.07	242.42	0.99	0.32

## Discussion

Various indicators can be used to assess the occurrence and intensity of factors that may have a negative impact on animal welfare. Nowadays, the welfare assessment mostly relies on evaluating the physical and mental health parameters (the so-called animal-based indicators) (34). Although a comprehensive, valid tool for assessing the welfare of cats in shelters, which could be used as a basis for the design of a protocol, is not currently available (1), the use of various health-oriented indicators (most commonly the body condition, coat condition and symptoms of upper respiratory tract infections) have been tested and described in studies assessing the quality of life of cats in various contexts (13, 31, 34, 37, 40, 41). In our study, we focused on documenting changes in eight health-oriented indicators of welfare (body condition, third eye visibility, eye discharge, eye irritation, nose discharge, the presence of pathologically induced respiratory sounds, coat condition, abnormal posture, and lameness) in group-housed cats in a no-kill shelter using a 5-point assessment scale.

Our results show that the condition of more than a half of the cats was not optimal upon their admission to the shelter. Among monitored indicators, the highest number of cats with a deteriorated condition was found in the body condition indicator. More than a quarter of all admitted cats were malnourished and almost 10% of cats were slightly overweight. However, 62.3% of cats had an optimal body condition, which is a result comparable to that reported by Marston and Bennett (42) who found 70.6% of cats admitted to a shelter in an optimal condition. In our study, during the stay in the shelter, the scores improved in 24% of cats. On the other hand, there was a permanent deterioration of the body condition in 8% of the cats during the stay. In another 9.6%, there was a temporary change in the body weight (first there was an improvement, then deterioration, or vice versa). Changes in body condition may occur in animals that are lactating or pregnant; however, no such cat monitored during the study period. For group-housed cats, monitoring whether each individual has ingested a sufficient amount of food can be problematic; the number of resources (in this case food bowls) is recommended to be adapted to the number of cats plus one more should be provided (*n* + 1 rule, where n is the number of cats) (43). This recommendation was not followed in the shelter where the data collection took place. Other factors that negatively affect the food intake may include the presence of passive aggression among the cats stemming from situations where the animals prevent each other from accessing resources (1). The resources must therefore be located in various places throughout the housing area. In the monitored shelter, there were five places where bowls with dry food were always accessible to the animals. Wet food was provided at five other places twice a day. In facilities where the distribution of different types of food is not uniform, there may be differences in the structure of the rationed feed among the animals that always have access to the source and animals that have a limited or no access to it, as other individuals prevent it. Reduced food intake in cats is associated not only with social conflicts but with stress in general (30). However, in most animals, stress decreases with the increasing LOS in the shelter (cats usually adapt to the shelter environment within 2–5 weeks) (44). Kessler and Turner (25) studied 140 cats staying at a boarding cattery for 2 weeks. Pronounced reduction of level of stress was found in the first 4 days, two–thirds of the cats adapted within 2 weeks. However, cats which introduced into communal pens at an animal shelter showed not only significant behavioral changes during the first 4 days, but changes were seen throughout the entire first month as the cats adapted (44). We believe that the stress caused by placement in the new environment may have played a role in those animals whose body condition deteriorated in the first few weeks after admission to the shelter monitored in our study, but probably no longer played a significant role in animals that showed a deterioration after several weeks of stay (i.e., animals that are expected to have a higher degree of adaptation to the environment than newly admitted cats). On the other hand, there is a great variability in how cats perceive and react to stressful stimuli (45). In the case of chronic stress, it may take a long time for a change in body condition to manifest. Whereas, no effect of sex and the LOS of cats on the body condition was found in our study, the effect of cats' age on the body condition was significant; a higher age was associated with an increased score. The decreased activity in older cats may potentially contribute to a weight gain (46), resulting in higher body condition scores. In addition, a loss or a decrease in appetite may occur as a result of a disease or infection, e.g., URTD, a common health problem occurring in shelter cats (14). A young age of cats was also found to be a predictor for the URTD development by Binns et al. (47). However, in our study no correlation was found between the body condition and signs of URTD although in some animals the decrease in the body condition score occurred simultaneously with the increase in the score in the indicators related to URTD. This result is consistent with the findings of Tanaka et al. (13); they reported a weight loss not to be directly associated with the development of URTD in shelter cats although the tendency for URTD to develop was 5.6 times higher in cats with high levels of stress, causing a reduction in food intake. Stress is known to be a risk factor for the development of an infection as it can inhibit the production of mucosal antibodies, especially immunoglobulin A (S-IgA) (12).

After the quarantine period, the animals were placed in a group among other cats. Although the vaccination of shelter cats against calicivirus and herpesvirus was routinely performed while in quarantine, it should be noted that in the case of herpesvirus, there is no FHV-1 vaccine that can protect against an infection with virulent virus (48). The infection may lead to the virulent virus becoming latent with the possibility of reactivation during periods of severe stress. Thus, the reactivated virus may cause clinical symptoms even in vaccinated animals or it can be excreted. The vaccination against calicivirus does not prevent the infection, although in many cases it can alleviate severe symptoms. The existence of many strains of calicivirus has been confirmed (49). The transmission of infections is very likely as cats in a group are in a direct physical contact with each other, they share the common area, litter trays, food, and water bowls. However, URTD is a common problem even in shelters where cats are housed individually.

In a study by Pedersen et al. (50), only 4% of cats were found to excrete herpesvirus upon arrival at the shelter. After 1 week, its excretion was detected in 52% of cats. In the case of calicivirus it was in 15% of cats (50). In our study, during the cats' stay in the shelter, an increase in scores in indicators related to URTD occurred in 8.8–18.4% of cats (the highest number of cats with a deteriorated score was recorded for the eye discharge indicator, the lowest for the nose discharge indicator). The LOS in the shelter positively predicted a higher score of the CCI. This finding is likely to indicate a mutual transmission or an activation of pre-existing latent infections in cats due to stress resulting from the shelter environment.

The visibility of the third eyelid is another indicator that may be associated with the occurrence of URTD—a third eyelid may be visible due to conjunctivitis (51). A significant correlation between the third eyelid visibility and the URTD symptoms was found in our study. In almost all cats, the cause of the appearance of the third eyelid was URTD. In addition to URTD, the third eyelid usually occurs also due to the presence of an ocular, neurological or systemic disease (52). Abnormal protrusion may occur in both or only one eye. While an ocular problem usually affects only one eye, a systemic disease is usually presented in both eyes. The visibility of the third eyelid can be used as a subtle indicator of a worsened condition of a cat as it can develop in connection with a weight loss or dehydration (52). However, in our study the mean scores of the third eye visibility indicator and the mean body condition scores were not correlated.

The coat condition may be adversely affected by food quality, chronic illness, presence of social conflicts and changes in normal behavior (insufficient or, conversely, excessive care of the coat (53). Also, the number of animals in the group seems to be connected to the condition of the cats' coat. The connection was found both in shelter cats (34) and in feral cats in colonies (40). The coat condition was not optimal only in 15% of the animals admitted to the shelter monitored in our study. More than a half of these cats were rated with a score of 3. This finding differs from Zito et al. (41). However, the higher percentage of cats with optimal condition found in our study may be explained by the fact that Zito et al. (41) focused on the monitoring of free roaming cats. In the cat population monitored in our study, some animals were brought to the shelter by their original owner. The number of these cats is, however, unknown. The shelter database, which provided us with additional information on cats, did not contain any information on the origin of admitted cats.

Arhant et al. (34) described the association of cats' prolonged stay in a shelter with an increase in the percentage of cats with deteriorated coat condition. However, in our study, the LOS did not predict the deterioration of the coat condition. In most cats admitted with a suboptimal score of the coat condition, the condition of the coat improved during their stay in the shelter. This finding may reflect the regular intake of quality food and adequate veterinary care in the shelter (application of antiparasitics, initiation of treatment related to the condition of fur and skin) and indicate a low incidence of serious social conflicts in the group. Although recording of the interactions between cats was not the subject of our study, aggravated conflicts manifested by fighting that could result in skin damage were not obvious in the monitored population. In our study, the association between the occurrence of the URTD signs and the coat condition deterioration was found, which is in agreement with the results of a study by Gilhofer et al. (40). In their study, a half of the cats with an impaired coat condition also had the symptoms of URTD.

Abnormal posture and lameness were also monitored in the population of shelter cats in our study. Most of the cats (94.5%) admitted to the shelter showed no signs of impaired mobility or/and lameness. The score improvement during the shelter stay in cats with the initial scores higher than 1 was found, which was the result of the treatment (or surgery) after admission. Injuries were the main causes of lameness and impaired mobility in admitted cats. Permanent deterioration of the score during the shelter stay was documented in only one cat.

TerWee et al. (54) documented a connection between lameness and the presence of URTD. Some calicivirus strains may cause pain-induced limping in infected cats. In our study, no correlation was found between the mean scores of abnormal posture and lameness indicator and the mean scores of other indicators. The abnormal posture and lameness score was, however, linked to the cat's LOS in the shelter, as the LOS predicted higher scores in this indicator. The results suggest that the cats with worsened scores spent more time in the shelter, which can be explained by the reduced adoption potential of these animals. The cats' health was found to be a significant factor affecting the adoption preferences of potential new owners of shelter animals (55).

Overall, 41.6% of cats (*n* = 52) experienced a deterioration in at least one of the eight health indicators during their stay in the shelter monitored in our study. The prolonged stay is an issue especially in those shelters where it is not allowed (due to the compliance with the legislation or ideological reasons) to euthanize unwanted animals. Therefore, the efforts of the shelter operators should be focused as much as possible on strengthening the factors that can reduce the stay to a minimum, resulting in a successful adoption or the return of the animal to its original owner. If the shelter staff cannot select animals and create smaller stable groups due to a lack of space, as was the case of the facility monitored in our study, housing a large number of cats in one group is a risk factor contributing to the development and spread of diseases (19), in spite of consistent quarantining of the admitted individuals. In the Czech Republic, specific requirements concerning the design, equipment, and management of shelters are not covered by the legislation on animal protection and veterinary care. There are only some recommendations available, but these are not legally enforceable by control authorities. Animal shelters can therefore be established in facilities not optimally adapted to such purpose from the viewpoint of disease control. In the shelter monitored in our study, due to a lack of space, the quarantine section of the shelter was not completely separated from other areas where non-quarantined cats had access. The effectiveness of such quarantine is thus significantly diminished. From the viewpoint of survival of pathogens, other sections of the shelter also seemed problematic (e.g., inappropriately chosen, lacking an easily-washable floor in the outdoor pen and overcrowded housing area, consisting of a lot of hiding spots made of various materials not easily washable). Although these aspects of the environment meet the behavioral needs of cats and help reduce stress (56), on the other hand, they make sanitation difficult and do not allow a high standard of hygiene to be maintained, which is extremely important in a multi-cat environment. Pathogens can thus remain in the environment for a long time, in the case of feline parvovirus or oocysts of *Isospora* even for months to years (57).

Although the primary aim of this study was to detect changes in health-oriented welfare indicators of shelter cats, we also had the opportunity to verify the practicality of the 5-point assessment scale during the data collection. This scale could account even for small changes in the condition for the given indicator. As such, its use during monitoring appeared beneficial. On the other hand, the decision-making process between the individual scores was sometimes problematic. In spite of the use of a calibration photo sheet, this was especially true for the indicators of eye discharge, eye irritation and nose discharge. Animals were assessed only visually and acoustically without handling which in the case of presence of pathologically induced respiratory sounds indicator may appear to be a disadvantage, as if the animal is observed from a distance, the early stages of developing symptoms may not be well-recognized. To maintain the same scoring system for all indicators and due to the practicality of use, the 5-point scale was chosen also for the body condition assessment instead of the frequently used 9-point scale. Although the differences between the individual scores are more noticeable in the 5-point scale, the disadvantage of its use includes the inability to record more subtle changes in the body condition. In contrast to some studies (13, 56), exact body weight measurements were not carried out in our study, thus minor changes in the body weight could not be observed due to the approach taken (the 5-point body condition assessment scale). To detect a change with this scale, the amount of the cat's body fat had to change by at least 7%, as approximately this percentage represents the difference between the individual scores of the scale (36). The overall disadvantage of assessing animal-based indicators is their subjectivity (25). In our study, the cats were assessed by two trained evaluators, one of whom was a caretaker of the shelter cats. The involvement of the person directly responsible for the care of the animals in the assessment is advantageous, as this person has an insight into changes in the condition of the animals. However, the participation of another (trained but impartial) person is necessary so as not to distort the results.

Limitations of this study related to the assessment of cats include the absence of formal intra-rater reliability calculation for the scale as both observers assessed the cats at the same time. Another drawback is the fact that due to the lack of records on the origin of cats, it was not possible to take this variable into account in the analyzes.

The low number of cats remaining in the shelter monitored in our study for more than 2 weeks may have influenced the results. Only three cats remained in the shelter for 26 weeks (these cats were evaluated 14 times, which was the maximum number of assessments recorded in our study). It can be problematic to collect a sufficient amount of data to evaluate the effect of the long-term housing on changes in indicators in shelter cats, as most animals terminate their stay in a shelter earlier. Nonetheless, the cats' LOS in the shelter should be reduced to a minimum and we support the efforts of the facilities to implement procedures that lead to this goal.

In case of significant fluctuations in the total number of cats housed in the shelter during the monitored period, it would be appropriate to consider the differences between numbers of cats and thus population density in the shelter as a factor that may also affect the results of individual assessments. However, the number of cats housed in the shelter during the monitored period was stable over time and the difference in the number of animals present during individual assessments was not significant.

## Conclusions

Cats admitted to shelters may show various signs of deterioration in the health condition. In our study, this was the case in more than a half of the admitted cats. Forty-one percent of cats experienced a deterioration in at least one of the health indicators during their stay in the shelter. The results suggest that despite providing basic needs, there is still a risk of disrupting the health of cats in the shelter environment. Moreover, housing cats in one large group can be problematic in terms of disease control and prevention.

## Data Availability Statement

The original contributions presented in the study are included in the article, further inquiries can be directed to the corresponding author/s.

## Ethics Statement

Ethical review and approval was not required for the animal study because no experimental procedures were performed. All data on the shelter cats analyzed in this study have been obtained strictly by visual observation of the cats and from the shelter records. The cats were housed and provided with care by the shelter personnel in accordance with the current animal welfare and veterinary legislation.

## Author Contributions

VVo and EV: conceptualization, data curation, and methodology. EV and VVe: supervision. VVo: writing—original draft. EV and VVe: writing—review and editing. All authors contributed to the article and approved the submitted version.

## Funding

This study was supported by ITA VETUNI (Project No. 2021ITA22).

## Conflict of Interest

The authors declare that the research was conducted in the absence of any commercial or financial relationships that could be construed as a potential conflict of interest.

## Publisher's Note

All claims expressed in this article are solely those of the authors and do not necessarily represent those of their affiliated organizations, or those of the publisher, the editors and the reviewers. Any product that may be evaluated in this article, or claim that may be made by its manufacturer, is not guaranteed or endorsed by the publisher.

## Footnotes

^1^Note that as this was not the main aim of the study and models with an interaction would not improve the fitness of the model; the interactions among the variables were not included into the analysis.
